# Functionalized UiO-66-NH_2_ by trimellitic acid for highly selective adsorption of basic blue 3 from aqueous solutions

**DOI:** 10.3389/fchem.2022.962383

**Published:** 2022-09-02

**Authors:** Tingting Wang, Lin Han, Xin Li, Tianen Chen, Shifeng Wang

**Affiliations:** ^1^ Innovation Laboratory of Materials for Energy and Environment Technologies, Tibet University, Lhasa, China; ^2^ Hofmann Institute of Advanced Materials, Shenzhen Polytechnic, Shenzhen, China; ^3^ Institute of Oxygen Supply, Everest Research Institute, Tibet University, Lhasa, China; ^4^ Key Laboratory of Cosmic Rays (Tibet University), Ministry of Education, Lhasa, China; ^5^ School of Chemical Engineering, University of Science and Technology Liaoning, Anshan, Liaoning, China

**Keywords:** basic blue 3, UIO-66-NH2, adsorption, dye, metal-organic frameworks

## Abstract

A novel metal-organic framework (MOF) UiO-66-TLA (UiO-66-Trimellitic Acid) was synthesized by one-pot method with trimellitic acid as modifier, which can effectively remove the basic dye Basic Blue 3 (BB3) in wastewater. Modification with carboxyl groups facilitates the adsorption of the cationic dye Basic Blue 3. The adsorption of BB3 by the modified UiO-66-TLA was significantly greater than that of its parent MOF. The adsorption capacity of the modified UiO-66-TLA for BB3 (234.23 mg g^−1^) was 93.2% higher than that of the original UiO-66-NH_2_ (121.24 mg g^−1^), this is closely related to the electrostatic interaction of -COOH in trimellitic acid. UiO-66-TLA was successfully synthesized as indicated by various characterization results. The adsorption kinetics conformed to the pseudo-second-order model, and the adsorption isotherm conformed to the Redlich-Peterson isotherm. This indicates that BB3 is a multi-parameter model of monolayer/multilayer arrangement on the adsorbent surface, and its rate-controlling step is chemisorption. The adsorption process was non-spontaneous and belonged to an endothermic reaction, in addition, it has great adsorption stability and regeneration The interaction of the modified UiO-66-TLA with BB3 was mainly affected by mechanisms, such as electrostatic interaction, π–π stacking as well as the abundant functional groups on UiO-66-TLA surface. These results demonstrate that UiO-66-TLA is an efficient, regenerable, water-stable material for the removal of BB3 in solution, with practical implications, suggesting its potential as a dye adsorbent.

## Introduction

Metal-organic frameworks (MOFs) are crystalline coordination compounds whose periodic structures are composed of metal cations and clusters connected by organic linkers. (Barsukova et al., 2018). MOFs are one of the most widely investigated materials in the 21st century because of the customizability of their structures, their controlled porosity, and their high crystallinity. (Yin et al., 2018). MOF can be used for Gas separation and purification, (Yang et al., 2020), porous MOF glass, (Yin et al., 2022), electrochemical sensing, (Hatamluyi et al., 2020), membrane, (Wu et al., 2018), and photocatalytic. (Shi et al., 2021).

UiOs show better adsorption than other MOFs. Currently, some water-stable MOFs, such as UiO-66 and hierarchical porous UiO-66 (H-UiO-66), have been applied to the adsorptive removal of toxic chemicals from aqueous solutions. (Gao et al., 2020). Adsorption has the advantages of a low cost, high efficiency, great simplicity, and high efficiency. It also considered an environmentally friendly technique. (Zhang et al., 2019a). The application of functional groups, such as hydroxyl, carboxyl, and amino groups, can change the surface charge and electron distribution of the substrate to enhance the interaction between the adsorbate and the modified MOF. Li et al. (2021a) used UiO-66 s as a high affinity adsorbent to remove DCF and Cu(II) in an aqueous solution, and their respective adsorption capacities reached 385 and 52 mg g^−1^. Gao et al. (2020) reported an ultrahigh removal capacity of UiO-66-(COOH)_2_ (2,200 mg g^−1^) for rhodamine B, which was regenerative. After modification with -NH_2_, Chen et al. (2015) reported UiO-66-NH_2_ exhibited high adsorption capacity for cationic dyes. Furthermore, the porosity and high surface area of UiO-66-NH_2_ are favorable for adsorption and preconcentration of metal ions. (Guo et al., 2021). These works demonstrated that UiO-66-NH_2_ had a good removal capacity for harmful materials or heavy metal ions from wastewater.

Zhang synthesized zeolitic imidazolate framework-8 (ZIF-8)-loaded UiO-66-NH_2_ with significantly improved selective adsorption of methyl bromide and methylene blue. (Zhang et al., 2019b). UiO-66-NH_2_ @poly (sodium 4-styrenesulfonate, Nass) was synthesized to adsorb different dyes from wastewater, and the maximum adsorption capacity of methylene blue (MB) and basic fuchsine (BF) was 299.8 and 789.2 mg⋅g^−1^, respectively. (Zhang et al., 2022). To date, various kinds of nitrogen functionalized MOFs have showed high adsorption performances. The good adsorption performance is attributed to the variety of interactions afforded by the material, such as electrostatic interactions, hydrogen bonding, and π–π stacking. However, the use of UiO-66-NH_2_ for the adsorption of toxic dyes remains to be investigated.

It has been reported that hybrid frameworks with metal-nitrogen bonding are not a feasible solution for improving stability. (Cavka et al., 2008). Li et al. (1999) reported the synthesis of a MOF via metal carboxylate clusters, and the resulting material was crystalline and structurally stable by XRD analysis. Thus, trimellitic acid was added to UiO-66-NH_2_ to synthesize a novel and stable MOF containing both amino and carboxyl bifunctional groups. In general, the adsorbent will adsorb more anionic dyes after modification with -NH_2_, and more cationic dyes after modification with -COOH. Modification of UiO-66-NH_2_ with trimellitic acid can more effectively remove cationic dyes (basic dyes) in wastewater. Basic blue 3 (BB3) is used for dyeing wool/nylon and stick/acrylonitrile blended fabrics. It is also used for direct printing of acrylic carpets with rich colors and this basic dye has obvious toxicity, such as mutagenic and carcinogenic effects. (Li et al., 2021b). Therefore, it is necessary to remove it from wastewater. The–COOH groups make the surface of UiO-66-TLA negatively charged over a wide pH range, and its adsorption sites are well exposed to BB3 nanoparticles. (Gao et al., 2020). Zr-based MOFs exhibit extraordinary water resistance due to the strength of Zr-O bonds and unique geometry that prevents water inclusion and reduces hydrolysis reactions, (Ahmadijokani et al., 2021a; Ahmadijokani et al., 2013), both parent and functionalized UiO-66 also exhibit good structural stability. (Schoenecker et al., 2012). Furthermore, its nanoparticles are very stable and easily regenerated under the experimental conditions, and no significant reduction was observed even after 5 consecutive adsorption-desorption cycles. (Molavi et al., 2018; Ahmadijokani et al., 2020a; Ahmadijokani et al., 2020b; Ahmadijokani et al., 2021b).

In the present work, a novel and stable trimellitic acid-functionalized dual-ligand MOF material (UiO-66-TLA) was easily synthesized in bulk by a simple hydrothermal method, and its structure and morphologies were investigated. The cationic dye BB3 was adsorbed from the dye system with -COOH from the UiO-66-TLA sorbent. Through batch adsorption experiments, the effects of pH, time, initial concentration, and temperature on the adsorption process, as well as the adsorption kinetics, were investigated. The thermodynamics and adsorption isotherms were evaluated; the adsorption capacity of the adsorbent was calculated; and the adsorption mechanism was explored. Adsorption experiments were conducted to study the effects of adsorption isotherms, kinetics, thermodynamics, and pH, and the UiO-66-TLA material was characterized by SEM, TEM, EDS TGA, XRD, XPS, FT-IR, and BET.

## Experiments and methods

### Materials

Zirconium chloride (ZrCl_4_, 98%), N,N-dimethylformamide (HCON(CH_3_)_2_, DMF, 99.5%), 2-aminoterephthalic acid (C_8_H_7_NO_4_, >98.0%), trimellitic acid (C_9_H_6_O_6_, 96%), HCl (37.0%), methanol (CH_3_OH, ≥ 99.5%), glacial acetic acid (C_2_H_4_O_2_, HAc, 99.5%), basic blue 3 (C_20_H_26_ClN_3_O, BB3, 25%) were used in the experiments. All chemical reagents were analytical grade, and no further purification was required. Deionized water produced in the laboratory was also used.

### Synthesis of UiO-66-TLA

The synthesis of UiO-66-TLA was based on a previously reported one-pot hydrothermal method, with slight modifications. (Nanthamathee and Dechatiwongse, 2021). Briefly, ZrCl_4_ (0.3673 g) was dissolved in DMF (14.583 ml), and HCl (2.917 ml) was added, then the mixture was sonicated for 20 min first. Next 2-aminoterephthalic acid (0.3908 g, 0.002157 mol) and DMF (30 ml) were added in succession, which was sonicated for 20 min again. Subsequently, trimellitic acid (0.1578 g, 0.00075 mol) was added, and the mixture was sonicated for 20 min to mix uniformly. The solution was transferred to a 100 ml sealed bottle and heated in an oven at 80°C for 24 h under self-pressure. When the reaction was completed, it was cooled to room temperature, filtered, and dried. The product was purified several times with anhydrous methanol and DMF, and then dried at 80°C for use. It is worth noting that all the filtration steps was performed while the solution was hot, as the solubility of trimellitic acid is greater at higher temperature.

### Characterizations

The morphology, microstructure, and elemental composition of the samples were observed using field emission scanning electron microscopy (FE-SEM, SU8000, Hitachi), transmission electron microscopy (TEM, JEM 2100F, Japan), and energy dispersive spectroscopy (EDS). X-ray diffraction (XRD, BRUCKER D8 ADVANCE) was used to determine the phase composition of the samples. The valence states and distribution of chemical elements in the adsorbent were analyzed by X-ray photoelectron spectroscopy (XPS, 250Xi, Thermo Fisher) in the 2θ range of 5°–30°. The changes in the functional groups on the adsorbent surface were investigated by infrared (FT-IR, Nicolet Magna 560 E.S.P FT-IR spectrometer in the range of 4,000–400 cm^−1^ at resolution of 4 cm^−1^). The BET specific surface area and pore size distribution were determined using a nitrogen adsorption-desorption apparatus. The Zeta potential of the samples was measured at room temperature using Malvern Instruments Ltd. Dye ion concentrations were measured using UV-Vis spectroscopy.

### Adsorption experiment

All adsorption experiments were conducted in batch form. This study explored the adsorption performance of UiO-66-TLA for BB3 under different solution pH, reaction time, initial concentration, and temperature conditions. In a typical adsorption experiment, the adsorbent (30 mg) was added to an aqueous solution of BB3 (30 ml), and the resulting mixture was stirred in a thermostatic shaker. After adsorption, the clear filtrate was collected by suction filtration using a microfiltration membrane, and the concentration of BB3 was determined with a UV-Vis spectrophotometer. By setting different reaction times, the adsorption kinetics with an initial concentration of 300 mg/L were studied, and the adsorption equilibrium time was obtained. To explore the maximum adsorption capacity of the adsorbent, isotherm studies were conducted in the range of initial BB3 concentration of 100–500 mg/L. After shaking for 2 h, the remaining BB3 concentration in the supernatant was measured, and the adsorption capacity was calculated.

To study the adsorption kinetics of BB3 on UiO-66-TLA, 30 mg of UiO-66-TLA and 30 ml of 300 mg/L BB3 solution reacted for a certain time (2, 5, 10, 30, 60, 90, 120, 150, and 180 s). For the isotherm study, 30 mg adsorbent and 30 ml BB3 solution reacted for 2 h, and the BB3 concentration range was 100–500 mg/L. The data were fitted and analyzed by Langmuir, Freundlich, and Redlich Peterson models. To explore the adsorption of BB3 by UiO-66-TLA at different temperatures, the thermodynamic parameters were studied at 298, 313, and 328 K.

## Results and discussion

### Characterizations

**FIGURE 1:**
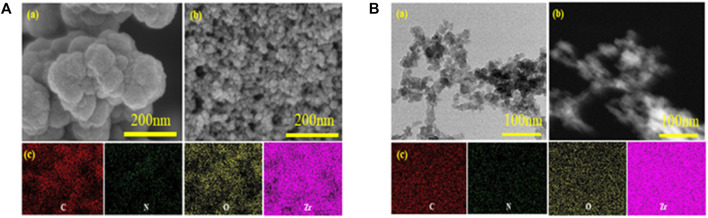
**(A)** SEM image of UiO-66-NH_2_ (a), UiO-66-TLA (b), and EDS mapping of UiO-66-NH_2_ (c); **(B)** TEM image (a), STEM image (b), and EDS mapping (c) of UiO-66-TLA.

SEM images of the UiO-66-NH_2_ and UiO-66-TLA are presented in Figure 1A. UiO-66-NH_2_ is a regular clustered octahedral particle, which is similar to previous reports. (Peñas-Garzón et al., 2022). The modified UiO-66-TLA had smaller particle morphology and relatively loose pores. Figure 1B shows the TEM image of UiO-66-TLA, where the regular particle morphology with a small amount of flocculation can be clearly observed, suggesting that UiO-66-TLA crystals are deformed on the exterior surface. This indicated that the UiO-66-TLA crystals were deformed on the outer surface, indicating the successful synthesis of the adsorbent.

**FIGURE 2:**
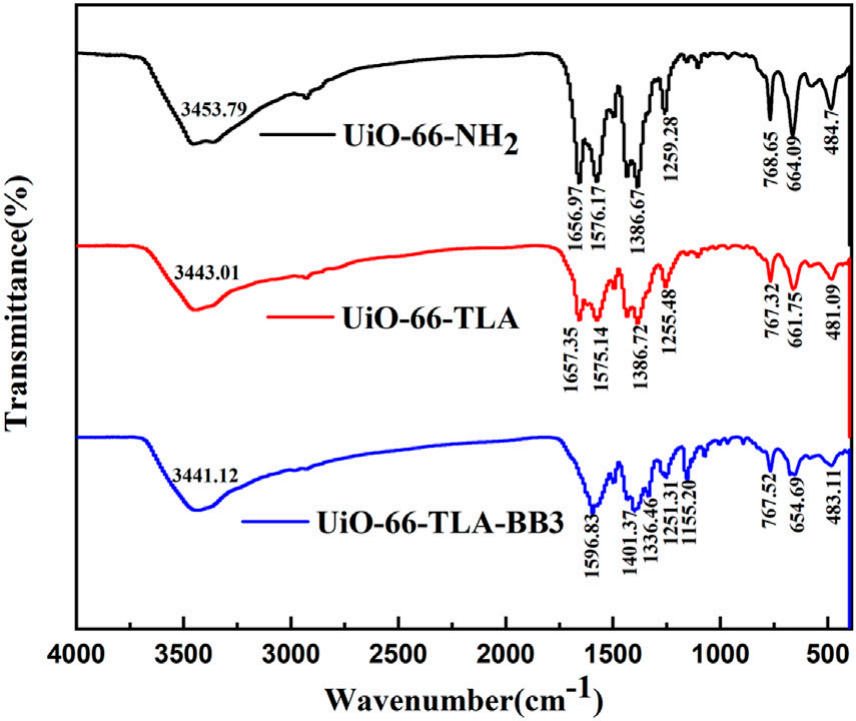
FT-IR of UiO-66-NH_2_, UiO-66-TLA, and UiO-66-TLA-BB3.

To further demonstrate the successful introduction of the ligand, the FTIR spectra were recorded and are shown in Figure 2. The main bands were 3,440 cm^−1^, 1,575 cm^−1^, and 1,255 cm^−1^, which were attributed to amino (ν(NH_2_)), carbonyl (ν(C=O)), and amino (ν(C-N)) stretching vibrations, respectively. (Wu et al., 2018). Supplementary Figure S1 shows the ^1^HNMR full-scan spectra of UiO-66-TLA, UiO-66-NH_2_ and trimellitic acid. The results show that UiO-66-TLA has two intense signal peaks at 8.0–8.25 ppm, which were aromatic hydrogen in trimellitic acid, and this peaks clearly implies that the modification was successful.

**FIGURE 3:**
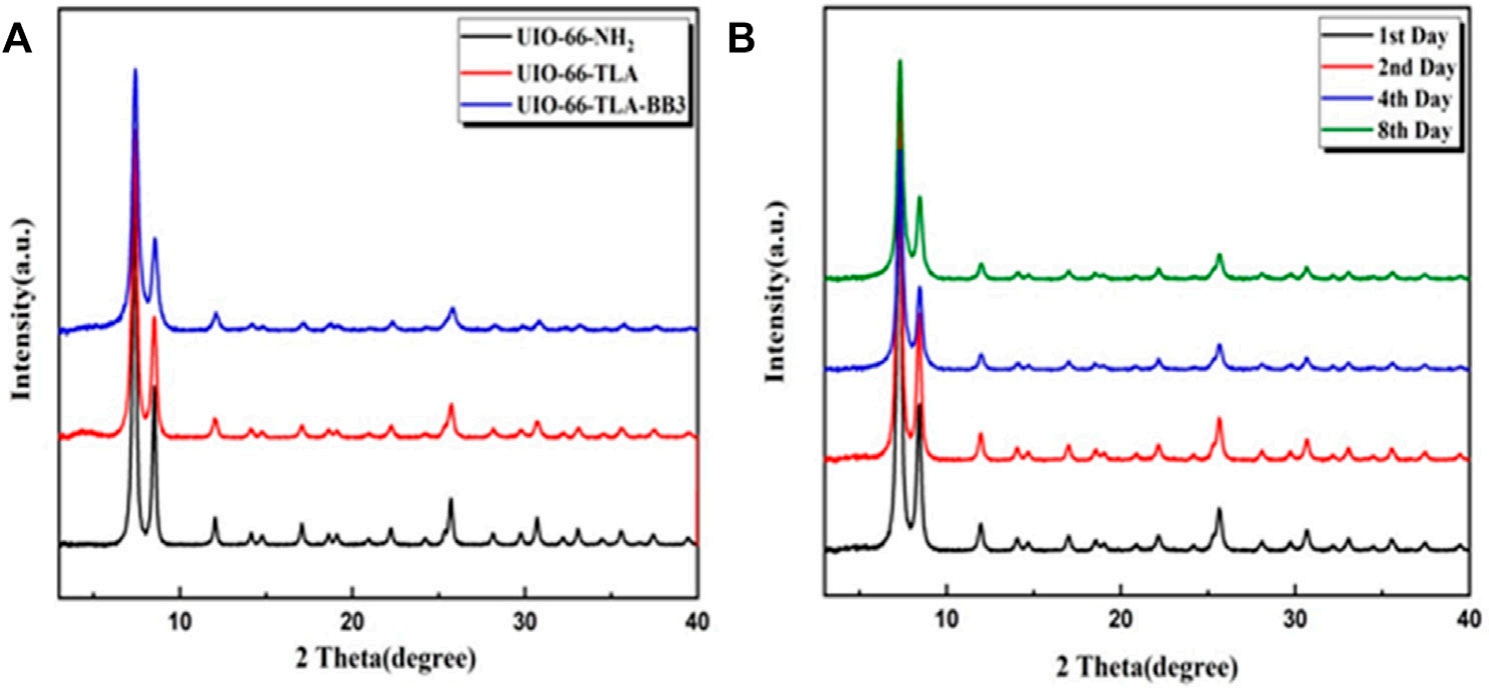
XRD spectra of **(A)** UiO-NH_2_, UiO-66-TLA, and UiO-66-TLA-BB3; **(B)** stability of UiO-66-TLA at 80°C for 1, 2, 4, and 8 d.

Figure 3A shows the XRD patterns of UiO-66-NH_2_ and UiO-66-TLA. The spectra have similar Bragg diffraction peaks, which are consistent with the diffraction peaks of UiO-66-NH_2_ reported by Pẽnas-Garźon. (Shen et al., 2020). This indicates that functionalization does not induce changes in crystal structure. The adsorbed UiO-66-TLA had the same characteristic peaks, indicating that electrostatic interactions had no significant effect on the crystal structure of UiO-66. From an economical point of view, the stability of the adsorbent is necessary for its application. As shown in Figure 3B, the XRD patterns of UiO-66-TLA were still intact after 1, 2, 4, and 8 d in the oven, demonstrating that UiO-66-TLA has good stability.

**FIGURE 4:**
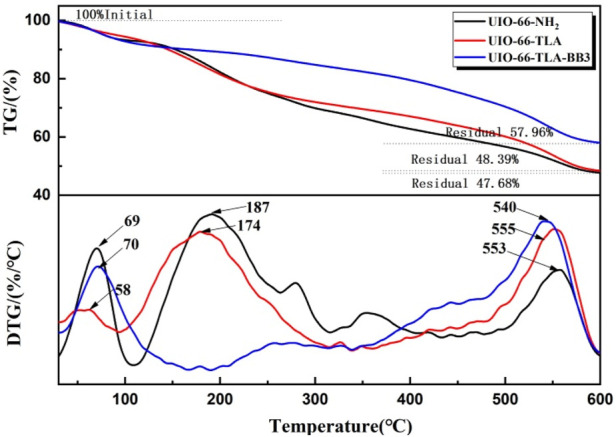
TG and DTG curves of UiO-66-NH_2_, UiO-66-TLA, and UiO-66-TLA-BB3.

Figure 4 compares the TGA and DTG curves of the three adsorbents to evaluate their thermal stability. The weight loss curves of the three adsorbents are similar, with 2–3 stages of weight loss with increasing temperature. Weight loss was observed up to 100°C and was attributed to the loss of physically adsorbed water molecules. (Wang et al., 2021a). The weight loss order of water was UiO-66-TLA > UiO-66-NH_2_ > UiO-66-TLA-BB3, which is closely related to the number of unsaturated metal centers. (Wu et al., 2018). When the temperature further increased to 187 and 174°C, the crystal structure was partially decomposed. When the temperature increased to 553, 555, and 540°C, the weight loss rates were about 33.41%, 32.66%, and 31.56%, respectively, which correspond to the thermal decomposition of ZrO(H_2_O)_1/3_C_8_H_3_NH_2_O_4_. (Cao et al., 2018). The mass fraction decreased stepwise, and the thermal stability increased gradually. (Sing, 1985).

**FIGURE 5:**
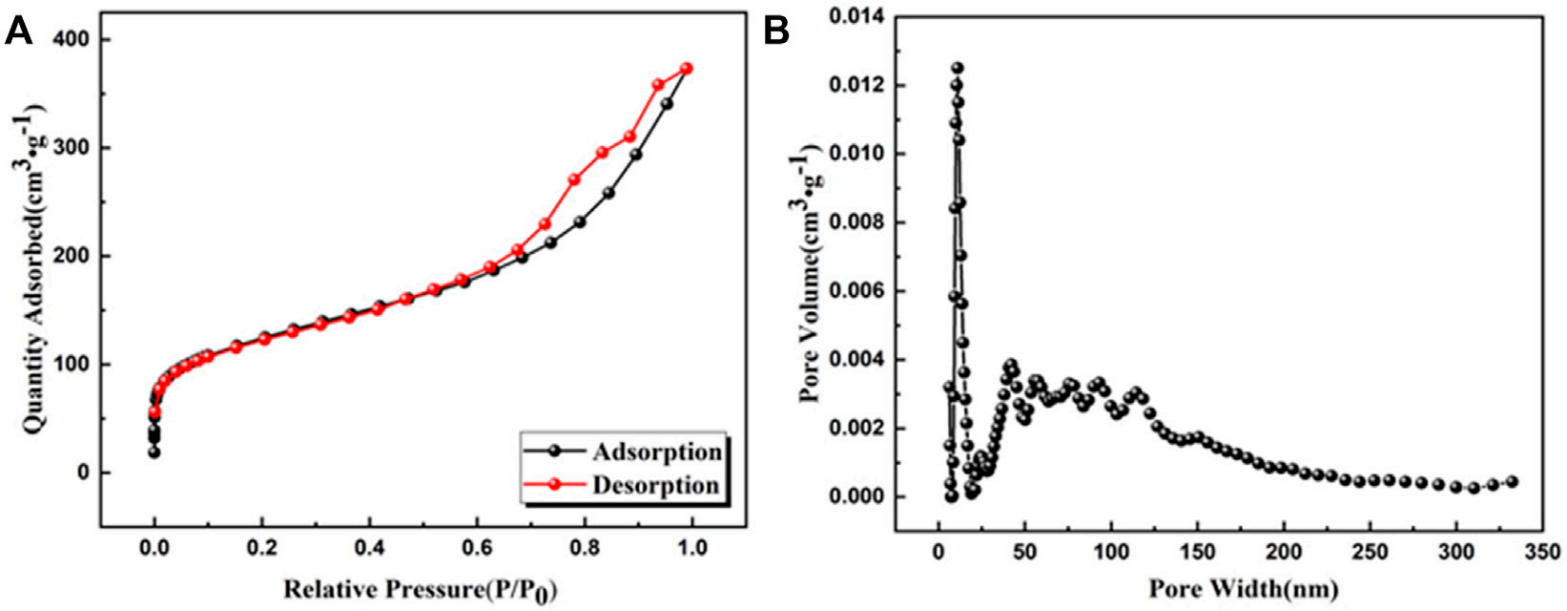
**(A)** N_2_ adsorption–desorption isotherm (77 K) and **(B)** DFT pore size distribution of UiO-66-TLA.

Nitrogen adsorption-desorption isotherms were tested to investigate the porous properties of UiO-66-TLA (Figure 5). UiO-66-TLA exhibited a Type II isotherm, which has a sharp increase in adsorption under lower pressure (P/P_0_ < 0.2). This indicates the adsorbent is a mesoporous material, with unrestricted monolayer-multilayer adsorption. (Wu et al., 2010). Compared with UiO-66-NH_2_ in Table 1, the surface area (437.419 m^2^/g), pore volume (0.529 cm^3^/g), and pore width (Mode) (10.960 nm) of UiO-66-TLA significantly decreased. (Shen et al., 2020).

**TABLE 1:** Porous features of UiO-66-NH_2_ and UiO-66-TLA.

Sample	Surface area (m^2^/g)	Pore volume (cm^3^/g)	pH_ZPC_
UiO-66-NH_2_	863	0.524	3.9
UiO-66-TLA	437.419	0.529	3.845

### Effect of time and adsorption kinetics

**FIGURE 6:**
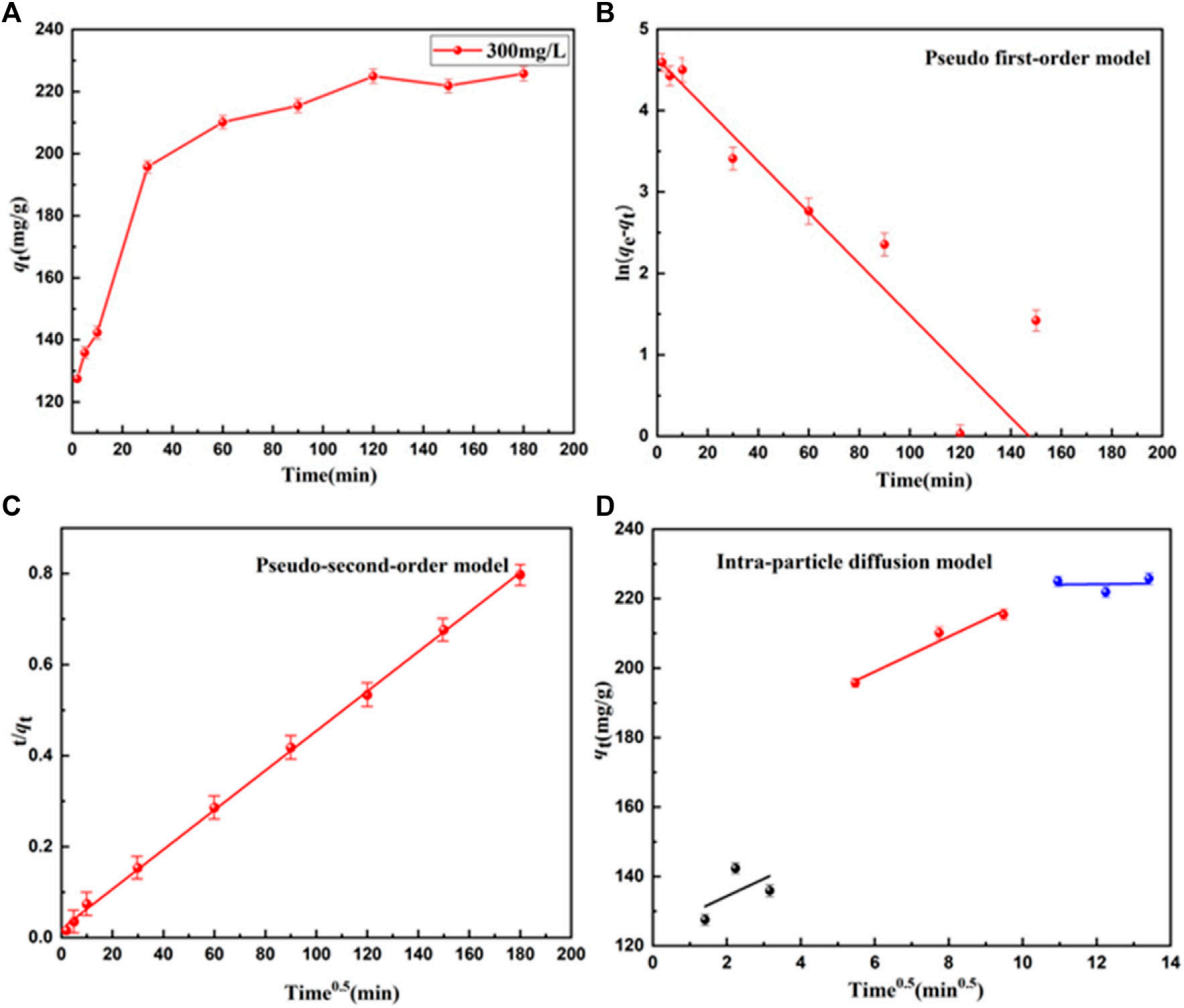
Effect of BB3 adsorption time on **(A)** UiO-66-TLA, **(B)** pseudo-first-order, **(C)** pseudo-second-order, and **(D)** intra-particle diffusion models.

Adsorption time is an important indicator of the performance of adsorbents. Adsorption time was used to further evaluate the adsorption kinetics of UiO-66-TLA. The adsorption process at each stage was simulated with an intra-particle diffusion model. The adsorption capacity of BB3 on UiO-66-TLA at different times is shown in Figure 6A. The adsorption capacity of UiO-66-TLA for BB3 increased rapidly within 60 min and reached the adsorption equilibrium within 120 min. The increase in adsorption rate is closely related to the adsorption sites on the adsorbent.

The kinetic parameters of different models were obtained by kinetic fitting of the experimental data in Supplementary Table S1. The fitting results of the pseudo-second-order model had a very high correlation coefficient *R* ^2^ (0.99916), which was significantly higher than the correlation coefficient *R* ^2^ (0.89058) of the pseudo-first-order model. Thus, the adsorption behavior of BB3 on UiO-66-TLA was more precisely described as chemical adsorption.

To further explore the intraparticle diffusion adsorption of BB3 on UiO-66-TLA, an intraparticle diffusion model was investigated. Figure 6D shows the relationship between q_t_ and t^1/2^ of BB3 on UiO-66-TLA, which was not linear over the course of the entire experiment. The trace was divided into three steps, indicating that the adsorption process was not affected by a single diffusion factor. Rather, membrane diffusion, intraparticle diffusion, and surface adsorption all contribute to the adsorption kinetics. Theirs slopes are expressed as K_1_ > K_2_ > K_3_, respectively. The adsorption sites and adsorption rate gradually decreased with time.

### Effect of concentration and adsorption isotherm

**FIGURE 7:**
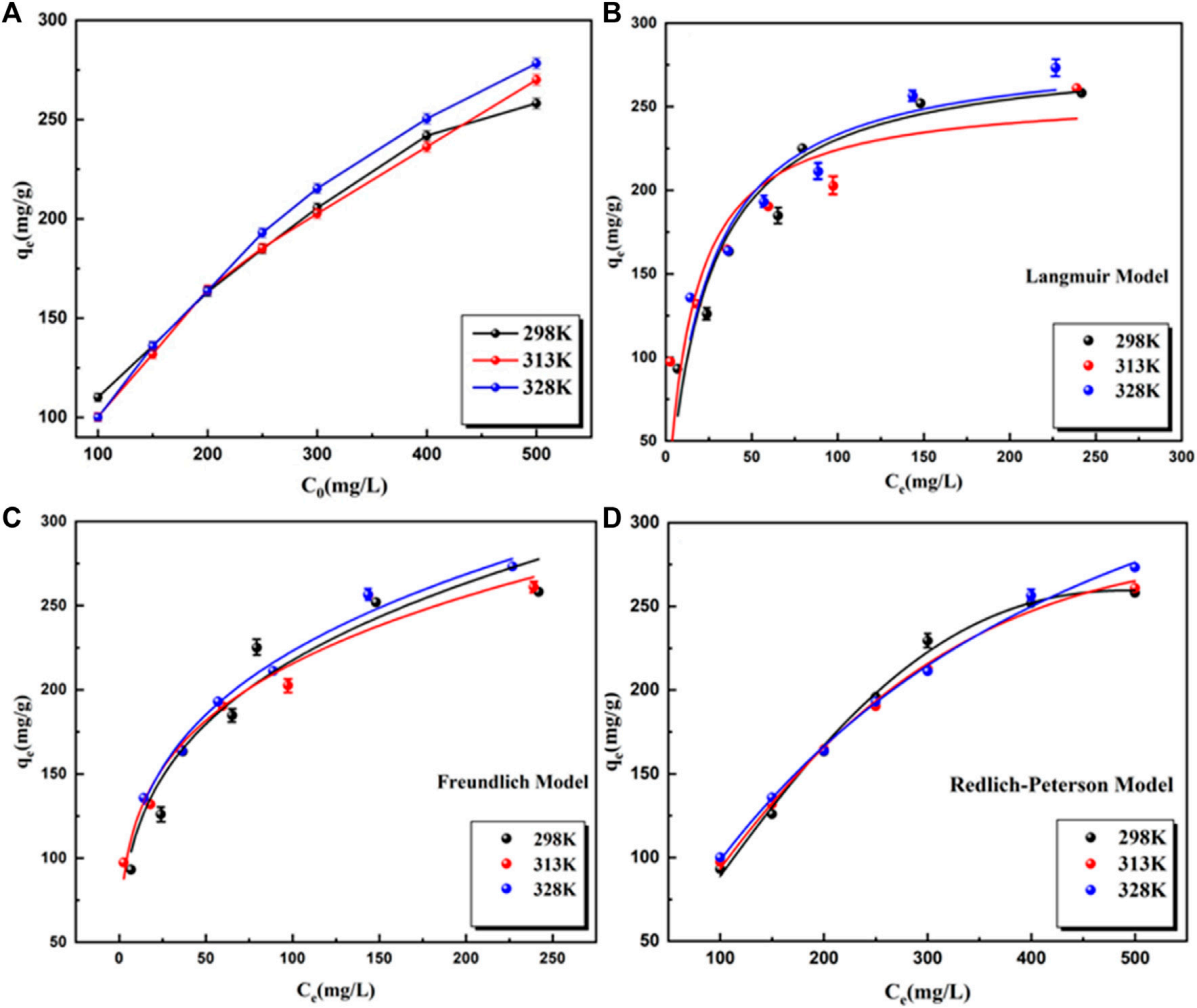
Effect of the initial BB3 concentration on the adsorption capacity of UiO-66-TLA at different temperature at 298 K **(A)**; Langmuir **(B)**, Freundlich **(C)** and Redlich Peterson **(D)**.

To explore the maximum adsorption capacity of BB3 on UiO-66-TLA, the initial concentration of BB3 was investigated. Figure 7 shows the adsorption capacity of UiO-66-TLA for BB3 at 298, 313, and 328 K. With the increase of the initial concentration of BB3, the adsorption capacity gradually increased until it reached saturation. This is because in a high concentration environment, the dye ions quickly occupy the adsorption sites, thus reaching the adsorption equilibrium. From the figure, the adsorption capacity of UiO-66-TLA for BB3 increased with increasing temperature, indicating the adsorption is an endothermic reaction. At 298 K, the maximum adsorption capacities of UiO-66-TLA and UiO-66-NH_2_ were 234.23 and 121.24 mg g^−1^, respectively. The effect of concentration and adsorption isotherm experiment of BB3 on UiO-66-NH_2_ has been done, and its related experimental data have been sorted into the supporting material (Supplementary Figure S3, Supplementary Table S4). This indicates the adsorption capacity of the modified UiO-66-TLA significantly improved. Table 2 lists several different BB3 adsorbents, and UiO-66-TLA was found to have the best adsorption capacity for BB3.

**TABLE 2:** Adsorption capacity comparison with various reported adsorbents for BB3.

Adsorbents	Maximum adsorption capacity (mg g^−1^)	Equilibrium time (min)	Temperature (K)	Reference
PVA-co-AAm/TiO2/SiO2 nanocomposites	140.9 mg g−1	420 min	298	Wawrzkiewicz et al. (2022)
Based on kraft lignin modified with ZrO2 and SiO2	83.5 mg g−1	60 min	—	Hassani et al. (2014)
Coal	84.72 mg g−1	10 min	298	Bartczak et al. (2022)
Cladium mariscus saw-sedge	44.29 mg g−1	60min	293	Muhammad et al. (2019)
Sawdust	28.69 mg g−1	60 min	293	Muhammad et al. (2019)
PANI/Fe3O4 composites	78.13 mg g−1	50–60 min	303	Marungrueng and Pavasant. (2006)
Palm fruit bunch particles	91.33 mg g−1	—	—	Karakuş et al. (2020)
Hydromagnesite stromatolite	15.72 mg g−1	15 min	293	Wang et al. (2021b)
UiO-66-TLA	234.23 mg g−1	120 min	298	This study

In this study, three isotherm models were selected to fit the data and to explore the interaction between BB3 and UiO-66-TLA. The results are shown in Figures 7B–D, and Supplementary Table S2. By comparing the correlation coefficient *R* ^2^ of each model, the larger the *R* ^2^ is, the more suitable. From the fitting results, the adsorption process of BB3 closely followed the Redlich-Peterson isotherm, indicating that each BB3 molecule is more likely to bind to the active site of the adsorbent in a multilayer form. When adsorbing macromolecules, there is a solid potential barrier between the pores and the adsorbate; therefore, the value of *α* is usually less than 1. (Shen and Gao, 2019).

### Effect of temperature and adsorption thermodynamics

**FIGURE 8:**
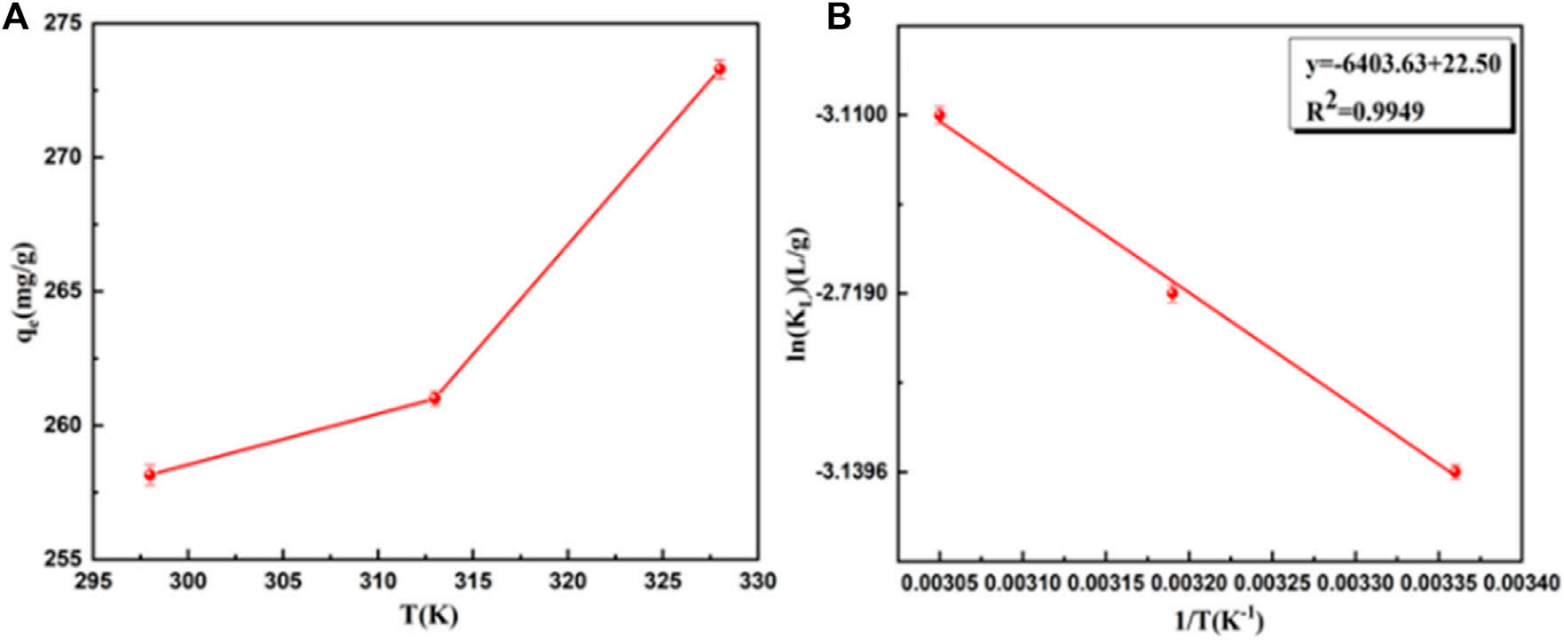
Effect of temperature on the adsorption of BB3 **(A)** and plot of lnK_L_ versus 1/T **(B)**.

The adsorption of BB3 decreased with increasing temperature, which confirmed an endothermic adsorption process. The thermodynamic study results and related parameters are shown in Figure 8 and Table 3. ΔG was positive at different temperatures, and the adsorption process of BB3 on UiO-66-TLA was non-spontaneous. In addition, the adsorption capacity decreased with increasing temperature. Furthermore, ΔH > 0 also indicates that the adsorption is endothermic and ΔS < 0 represents increased disorder at the solid–liquid interface. According to the literature that the physisorption energies are in the range of 0–20 kJ/mol, while the chemical sorption energies are between 80 and 400 kJ/mol, (Shen et al., 2018), it can be concluded that BB3 is chemically adsorbed instead of physical adsorption on UiO-66-TLA.

**TABLE 3:** Thermodynamic parameters for BB3 on UiO-66-TLA in a single-component system.

T	K_L_	ΔG° (kJ/mol)	ΔS° (J/(mol K))	△H° (kJ/mol)
298	0.04330	77.79	−8.159595311	53.475
313	0.06594	70.76	−22.60720899	
328	0.04460	84.82	−25.8582731	

### Effect of pH

**FIGURE 9:**
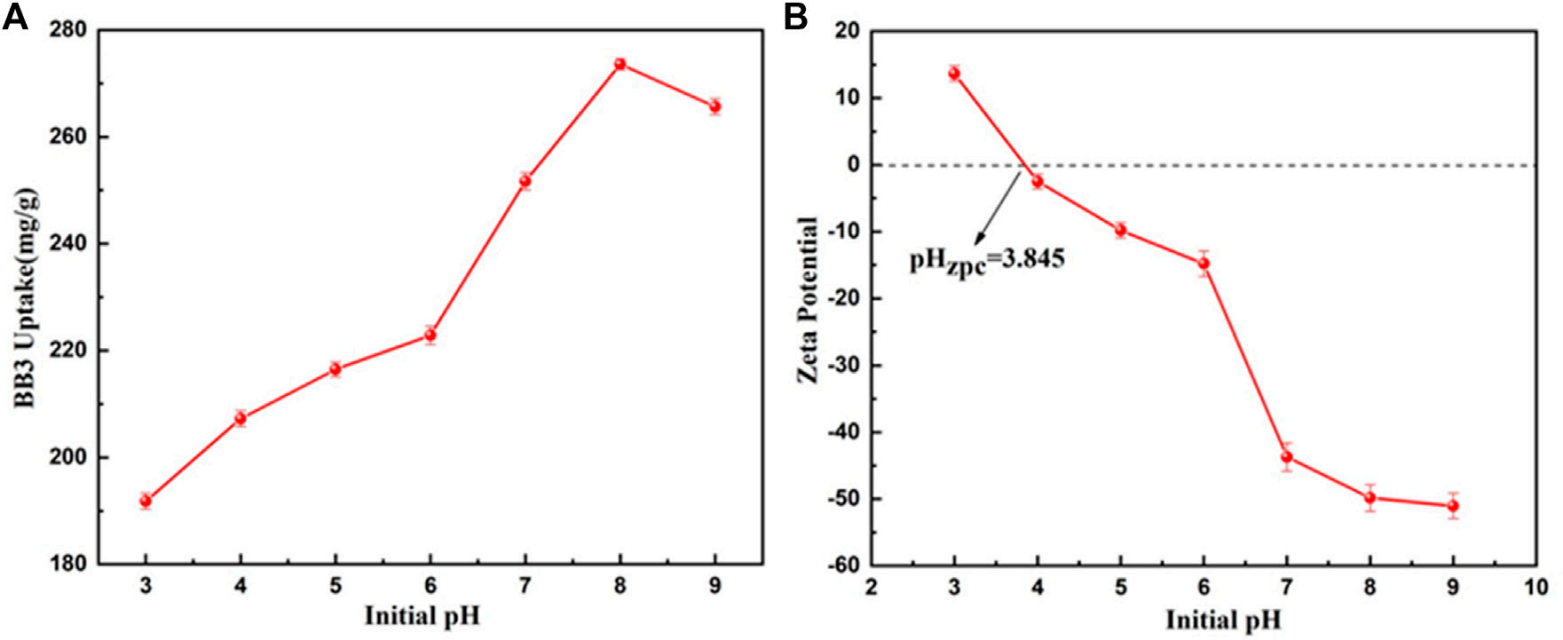
Effect of pH on the adsorption of BB3 **(A)** and zeta potential of UiO-66-TLA **(B)** (experimental conditions: BB3 = 300 mg/L, T = 298 K).

To further explore the practicality and adsorption mechanism of the adsorbent, Figure 9A shows the adsorption of BB3 by UiO-66-TLA at different pH (3–9) values. The adsorption capacity of UiO-66-TLA increased gradually with increasing pH, and the maximum was 273.62 mg g^−^1. As shown in Figure 9B, the pH value (zeta potential of 0 mV) of UiO-66-TLA was 3.845, and its zeta potential decreased with increasing pH. This may be due to the increase in its deprotonation degree. (Roulia and Vassiliadis, 2021). The surface of UiO-66-TLA was positively charged in a strong acid environment and negatively charged under alkaline conditions. The negatively charged surfaces of UiO-66-TLA (indicated by zeta potentials in Figure 9B) provide ideal affinity toward the BB3 cationic dye molecules. (Huang et al., 2022). Under these conditions, the electrostatic interaction between BB3 was the strongest and the adsorption capacity was the greatest.

### Selectivity of UiO-66-TLA

**FIGURE 10:**
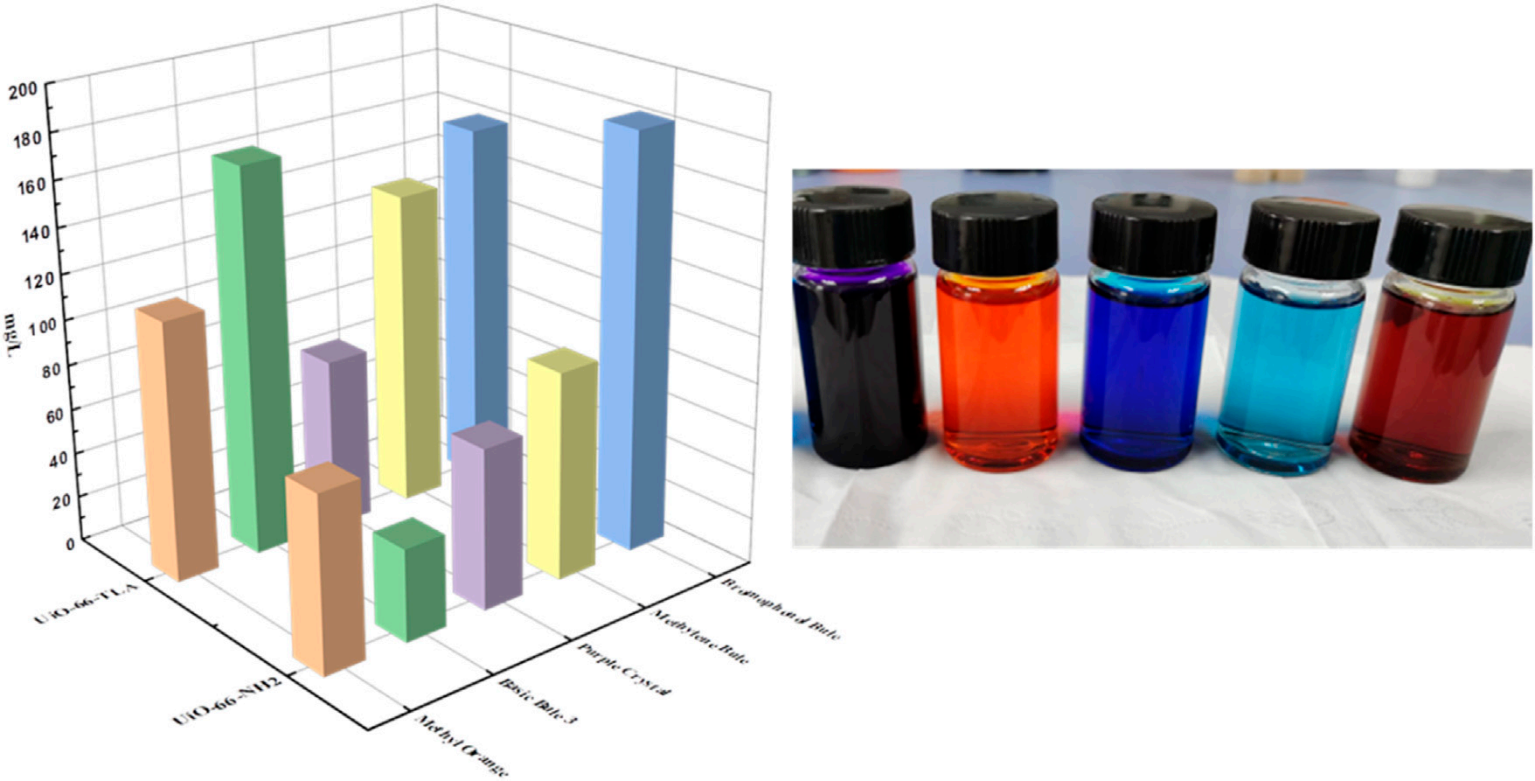
Selective adsorption of UiO-66-TLA.

The selective adsorption of BB3 by UiO-66-TLA in practical application was studyed by using laboratory wastewater, and a comparative experiment was carried out with the selectivity of UiO-66-NH_2_. The simulated wastewater contains methyl orange (MO), crystal violet (CV), basic blue 3 (BB3), methylene blue (MB) and bromophenol blue (BPB), and their concentrations are all 200 mg/L. Take 30 mg of adsorbent and 30 ml of waste liquid into a conical flask, shake for 2 h at room temperature, and then conduct solid-liquid separation. The dye ion concentration before and after adsorption was determined by UV-Vis. The Figure 10 show the adsorption results of UiO-66-TLA and UiO-66-NH_2_ for each dye in wastewater. By comparison, it is found that UiO-66-TLA in wastewater is selective to BB3, and the adsorption effect is better than that of UiO-66-NH_2_ before modification. The modified UiO-66-TLA contains carboxyl groups, which is beneficial to the adsorption of the cationic dye BB3. The modified adsorbent also has a certain adsorption effect on the anionic dye BPB, which may be due to its amino group. The carboxyl functional groups linked by chelation may occupy part of the site, making the adsorption effect partially weakened.

### Recycling of adsorbents

The reusability of the adsorbent is an important factor in water purification applications. The filtered adsorbent was washed 3 times with HCl for each adsorption. After drying under reduced pressure, the adsorbent is used for next cycle of dye adsorption. (Shen et al., 2021). There are overall four cycles, and the removal rate of BB3 is calculated based on the absorbance at 650 nm in each cycle. Although the removal rate of BB3 by UiO-66-TLA decrease in successive cycles (Supplementary Figure S2), after five cycles, the adsorption ability dropped by 17.45%. This demonstrates that UiO-66-TLA can remove BB3 effectively and keep the stable removal rate even after five cycles.

### Adsorption mechanism

**FIGURE 11:**
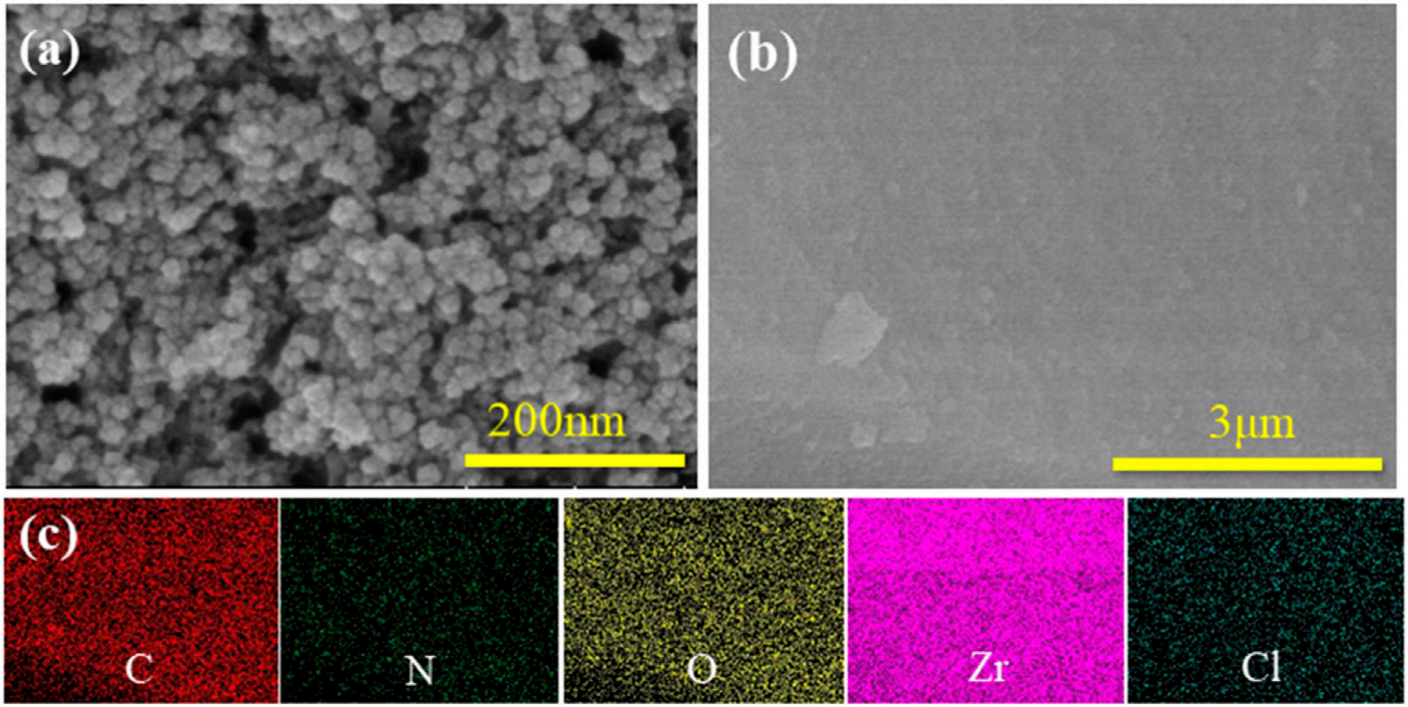
SEM **(A)** and EDS **(B,C)** images of the UiO-66-TLA-BB3.

The adsorption mechanism of BB3 on UiO-66-TLA was explored using SEM, TEM, EDS TGA, XRD, XPS, FT-IR, and BET on UiO-66-TLA after adsorption. The SEM images of UiO-66-TLA-BB3 (Figure 11) show dye particles attached to the surface and agglomeration. The EDS measurement results (Supplementary Table S3) show that the weight percentages of carbon and nitrogen in UiO-66-TLA-BB3 increased after adsorption, and there was a small amount of chlorine element, which indicates that the adsorbent can effectively adsorb BB3.

The FTIR spectrum of UiO-66-TLA-BB3 shows a peak at 1,155.20 cm^−1^, which was attributed to the alkyl C-N stretching vibration. (Adimule et al., 2021). The intensity of the absorption peak changed and the position shifted, indicating the atoms in the BB3 resonance structure are positively charged and attached to the carboxyl group. (Shen et al., 2021). In addition, the XRD pattern after adsorption is shown in Figure 3, and its characteristic diffraction peaks are consistent with those before adsorption, indicating that the crystal structure of the adsorbent in the solution is not destroyed.

**FIGURE 12:**
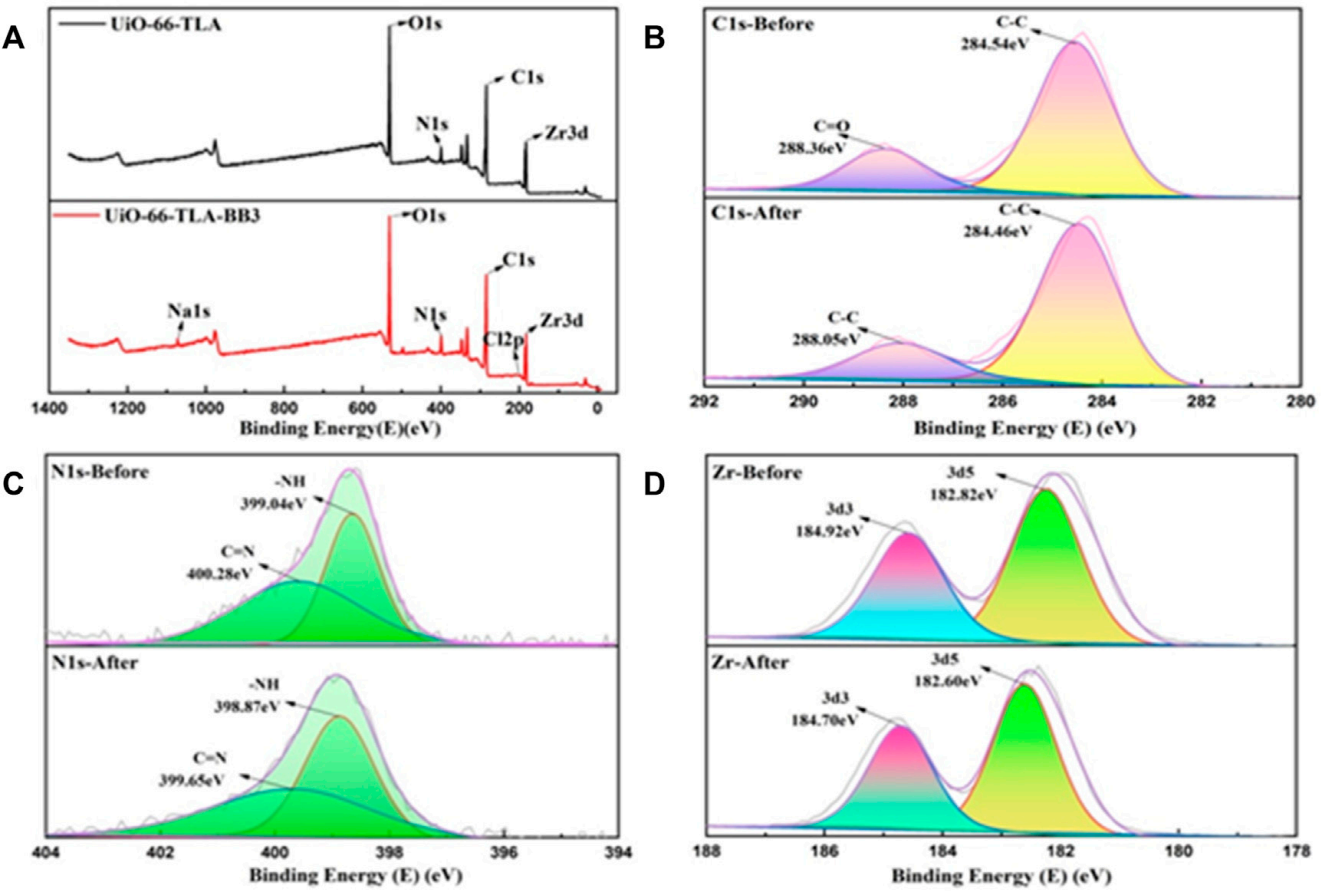
**(A)** XPS full-scan survey spectra of UiO-66-TLA and UiO-66-TLA-BB3, and high-resolution spectra of C 1 s **(B)**, N 1 s **(C)**, Zr **(D)** of UiO-66-TLA (before), UiO-66-TLA-BB3 (after).

The element energy changes before and after adsorption were measured by XPS to explore the mechanism of UiO-66-TLA adsorption of BB3. Figure 12A shows the total peaks before and after the adsorption reaction. UiO-66-TLA is mainly composed of C, N, O, Zr, and Cl elements, which remained after adsorption. The XPS peak of N1S is shown in Figure 12B, and its chemical states mainly include N-H (399.04 eV) and N=C (400.28 eV). After adsorption, N-H shifted to 398.87 eV, N=C shifted to 399.65 eV, and the binding energy decreased. There may be electrostatic interactions between adsorbents and cationic dyes, resulting in lower binding energies and lower electron densities. (Fu et al., 2015). As shown in Figure 12C, the XPS pattern of the 3 d region of Zr is a doublet, corresponding to 3d5/2 and 3d3/2. (Shen et al., 2021). After adsorption, the double peaks of UiO-66-TLA and UiO-66-TLA-BB3 shifted to the right, and the decrease in binding energy indicated a decrease in the electron density of the Zr clusters. These changes can be explained by the following assumptions: 1) the cationic dye can be adsorbed by the negatively charged UiO-66-TLA surface through electrostatic interactions; 2) since both UiO-66-TLA and the dye have aromatic rings, there may be π–π stacking interactions between UiO-66-TLA and BB3, resulting in a peak shift. (Xu and Ma, 2021; Han et al., 2022; Sun et al., 2022).

## Conclusion

A novel and stable metal-organic framework material, UiO-66-TLA, was prepared by one-pot method to remove basic blue 3 from aqueous solutions. UiO-66-TLA was characterized by FE-SEM, EDS, TEM, XPD, FT-IR, BET, and XPS analysis, and the effects of contact time, concentration, temperature, and pH on the adsorption efficiency were investigated. It was shown that UiO-66-TLA is an efficient material for the removal of BB3 in solution. At 298 K, the reaction reached the adsorption equilibrium within 120 min, and the maximum adsorption capacity was 234.23 mg g^−1^, compared with UiO-66-NH_2_ (121.24 mg g^−1^). Thus, the modification significantly improved the maximum adsorption capacity. The adsorption of BB3 by UiO-66-TLA conformed to the pseudo-second-order kinetics model and the Redlich-Peterson model, indicating that it is a chemical adsorption process. In addition, the adsorption of BB3 by UiO-66-TLA is a multilayer form. From thermodynamic studies, the adsorption capacity of UiO-66-TLA for BB3 increased with increasing temperature, indicating the adsorption is an endothermic reaction. The trimellitic acid-modified UiO-66-TLA makes the carboxyl group and the amino group exist at the same time, which is not only conducive to the adsorption of cations, but also has a good adsorption effect on anionic dyes. Electrostatic attraction and π–π stacking between BB3 and UiO-66-TLA were the predominant adsorption interactions. Furthermore, the crystal structure of UiO-66-TLA remained intact after being placed in water at 80°C for 8 days, with good reusability.

In conclusion, from the present findings, UiO-66-TLA is an efficient, regenerable, water-stable material for the removal of BB3 in solution, with practical implications, suggesting its potential as a dye adsorbent.

## Data Availability

The original contributions presented in the study are included in the article/supplementary material, further inquiries can be directed to the corresponding author.
