# Supplementary material for: Functionalized UiO-66-NH2 by trimellitic acid for highly selective adsorption of basic blue 3 from aqueous solutions
Source: Front Chem. 2022 Sep 2;10:962383. doi: 10.3389/fchem.2022.962383 (PMC9480502; doi:10.3389/fchem.2022.962383)
Supplement: Supplementary file 1 [file DataSheet1.docx]

**Supporting Information**

**Functionalized UiO-66-NH_2_ by Trimellitic Acid for Highly Selective Adsorption of Basic Blue 3 from Aqueous Solutions**

Tingting Wang^1,2,3,4^, Lin Han^1,2,3,4^, Xin Li^1,2,3,4^, Tianen Chen^2,5^, Shifeng Wang^1,2,3,*^

^1^ Innovation Laboratory of Materials for Energy and Environment Technologies, Tibet University, Lhasa 850000, China

^2^ Hofmann Institute of Advanced Materials, Shenzhen Polytechnic, Shenzhen 518055, P. R. China

^3^ Institute of Oxygen Supply, Everest Research Institute, Tibet University, Lhasa 850000, China

^4^ Key Laboratory of Cosmic Rays (Tibet University), Ministry of Education, Lhasa 850000, China

^5^ School of Chemical Engineering, University of Science and Technology Liaoning, Liaoning 114051, China;

*To whom correspondence should be addressed. E-mail: wsf@utibet.edu.cn

2.4. Adsorption experiment

The adsorbed amount q_e_ (mg/g) of the BB3 was calculated by equation (1)^1^:

$q_{e}=\frac{C_{0}-C_{e}}{m}V$ (1)

where q_e_ is the adsorption amount of adsorbent (mg/g), *C*_0_ and *C*_e_ are the initial and equilibrium concentrations (mg/L) of BB3, respectively; *m* is on behalf of the mass of adsorbent (g) and V is in the name of the volume of solution (mL).

To study the adsorption kinetics of BB3 on UiO-66-TLA, 30 mg of UiO-66-TLA and 30 mL of 300 mg/L BB3 solution reacted for a certain time (2, 5, 10, 30, 60, 90, 120, 150, 180 s). The obtained data was fitted with pseudo first-order (Eq.2)^2^, pseudo-second-order (Eq.3)^3^ and intra-particle diffusion (Eq.4)^4^ models. The calculation formula is as follows:

pseudo first-order Eq.2:

$\ln(q_{\text{e}}-q_{\text{t}})=\ln q_{\text{e}}-k_{1}t$ (2)

pseudo-second-order Eq.3:

$\frac{t}{q_{\text{t}}}=\frac{1}{k_{2}q_{\text{e}}^{2}}+\frac{1}{q_{\text{e}}}t$ (3)

intra-particle diffusion Eq.4:

$q_{\text{t}}=k_{\text{id}}t^{1/2}+C$ (4)

where k_id_ (mg g^-1^ min^-1/2^) is the rate constant. By plotting q_t_ against t^1/2^, a linear straight line can be obtained. The values of C and k_id_ are the intercept and slope of the line, respectively. The line graph of q_t_ versus t^1/2^ can be divided into three stages: the first stage represents the diffusion of the adsorbate to the outer surface of the adsorbent; the second stage represents the slow intraparticle diffusion process, and the third stage represents the equilibrium state. A higher correlation coefficient (R^2^) can indicate that the intraparticle diffusion process plays a role in the adsorption^5^.

The isotherm study is to take 30 mg adsorbent and 30 mL BB3 solution to react for 2 h, and the BB3 concentration range is 100–500 mg/L. It is fitted and analyzed by Langmuir, Freundlich and Temkin models.

The Langmuir isotherm can be described by Eq.5: assume monolayer adsorption on a uniform surface^6^.

$\frac{C_{\text{e}}}{q_{\text{e}}}=\frac{1}{K_{L}q_{\max}}+\frac{\text{C}_{\text{e}}}{q_{\max}}$ (5)

The Freundlich isotherm can be described by Eq.6: multilayer adsorption on uneven surfaces^7^.

$\ln q_{\mathrm{e}}=\frac{1}{n}\ln C_{\text{e}}+\ln K_{\text{f}}$ (6)

The Redlich-Peterson isotherm can be described by Eq.7^8^:

$q_{e}=\frac{AC_{e}}{1+bC_{e}^{g}}$ (7)

In order to explore the adsorption of BB3 by UiO-66-TLA at different temperatures, the thermodynamic parameters were studied at three temperatures of 298, 313 and 328 K, for example enthalpy change (ΔH, endothermic or exothermic process), entropy change (ΔS, the disorder of the system) and Gibbs energy (ΔG, the spontaneity of the adsorption process) changes are all used to describe the thermodynamic characteristics of the adsorption process (equation 8、9)^9,10^.

$\ln K_{L}=\frac{\Delta S^{\circ}}{R}-\frac{\Delta H^{\circ}}{RT}$ (8)$\Delta G^{\circ}=-RT\ln K_{L}$ (9)

where K_L_ (L mol^-1^) is the Langmuir constant. R is 8.3145 J (mol K)^-1^ and T is temperature (K)^11^. By plotting lnK_L_ against 1/T, the slope and intercept of the resulting line correspond to ΔH^。^and ΔS^。^respectively.

References

1. Yang W, Han Y, Li C, Zhu L, Shi L, Tang W, et al. Shapeable three-dimensional CMC aerogels decorated with Ni/Co-MOF for rapid and highly efficient tetracycline hydrochloride removal. Chemical Engineering Journal (2019) 375: 122076.

2. Rudzinski W, Plazinski W. Kinetics of solute adsorption at solid/aqueous interfaces: searching for the theoretical background of the modified pseudo-first-order kinetic equation. Langmuir (2008) 24(10): 5393-5399.

3. Regazzoni A E. Adsorption kinetics at solid/aqueous solution interfaces: on the boundaries of the pseudo-second order rate equation. Colloids and Surfaces A: Physicochemical and Engineering Aspects (2020) 585: 124093.

4. Li A, Deng H, Ye C, Jiang Y. Fabrication and characterization of novel ZnAl-layered double hydroxide for the superadsorption of organic contaminants from wastewater. ACS omega (2020) 5(25): 15152-15161..

5. Afzal S, Rahimi A, Ehsani M R, Tavakoli H. Experimental study of hydrogen fluoride adsorption on sodium fluoride. Journal of Industrial and Engineering Chemistry (2010) 16(1): 147-151.

6. Guo X, Wang J. Comparison of linearization methods for modeling the Langmuir adsorption isotherm. Journal of Molecular Liquids (2019) 296: 111850..

7. Ezzati R. Derivation of pseudo-first-order, pseudo-second-order and modified pseudo-first-order rate equations from Langmuir and Freundlich isotherms for adsorption. Chemical Engineering Journal (2020) 392: 123705.

8. Bolis V, Morterra C, Fubini B, Ugliengo P, Garrone E. Temkin-type model for the description of induced heterogeneity: CO adsorption on group 4 transition metal dioxides. Langmuir (1993) 9(6): 1521-1528.

9. Foo K Y, Hameed B H. Insights into the modeling of adsorption isotherm systems. Chemical engineering journal (2010) 156(1): 2-10.

10. Li Y H, Di Z, Ding J, Wu D, Luan Z, Zhu Y. Adsorption thermodynamic, kinetic and desorption studies of Pb^2+^ on carbon nanotubes. Water research (2005) 39(4): 605-609.

11. Hong S, Wen C, He J, Gan F, Ho Y. Adsorption thermodynamics of methylene blue onto bentonite. Journal of hazardous materials (2009) 167(1-3): 630-633.

12. Liu Y. Is the free energy change of adsorption correctly calculated?[J]. Journal of Chemical & Engineering Data (2009) 54(7): 1981-1985.


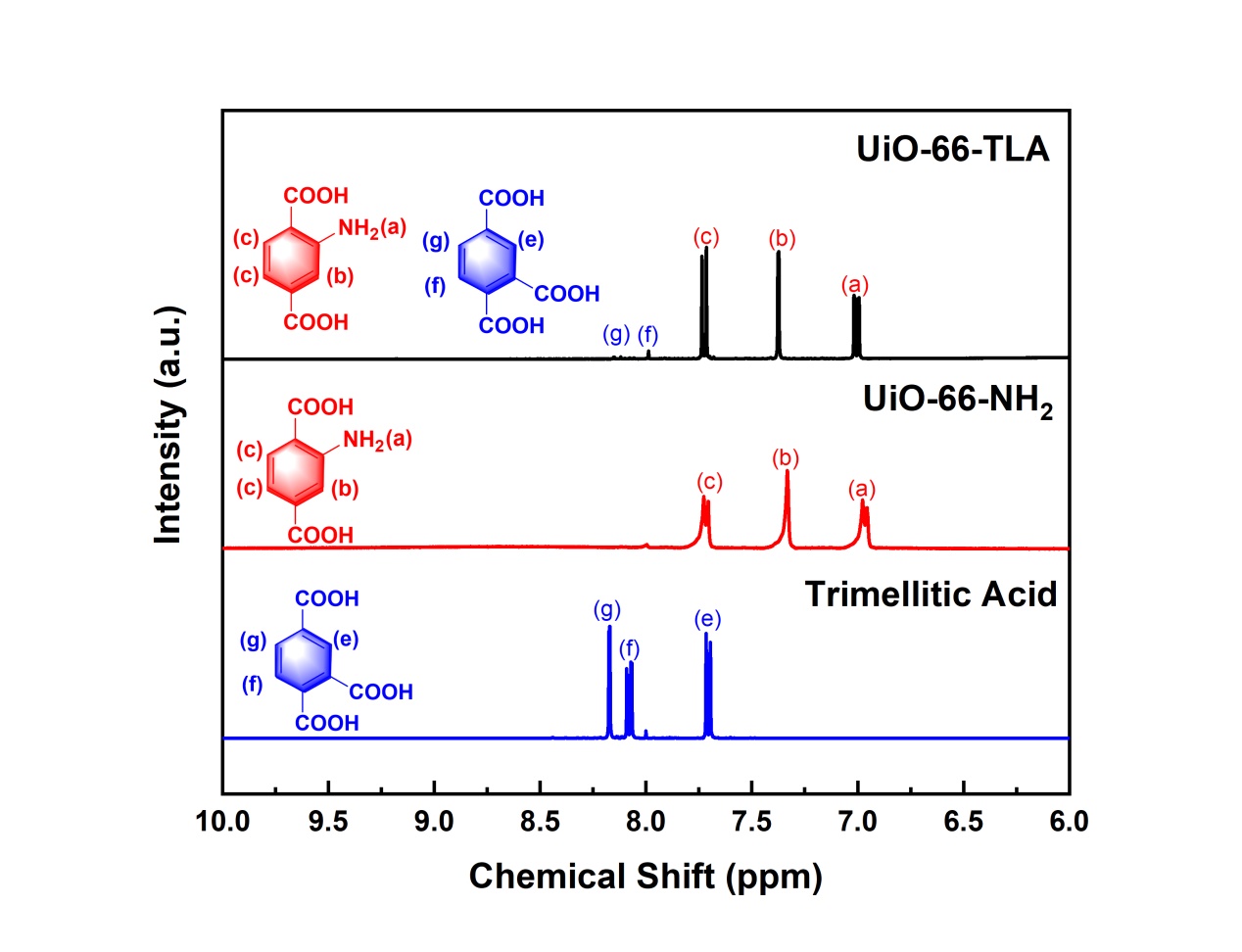


**Figure S1. ^1^HNMR spectra of UiO-66-TLA, UiO-66-NH_2_ and trimellitic acid.**


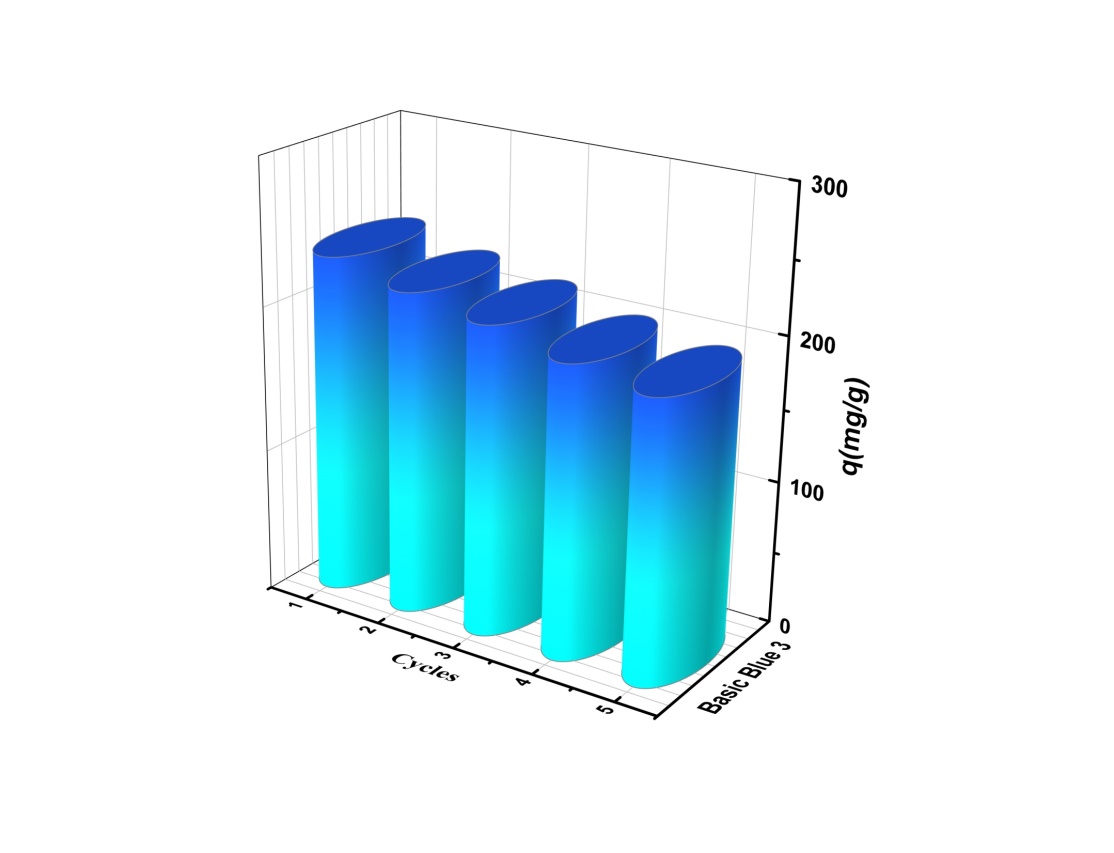


**Figure S2. Reusability of UiO-66-TLA in different cycles.**

**
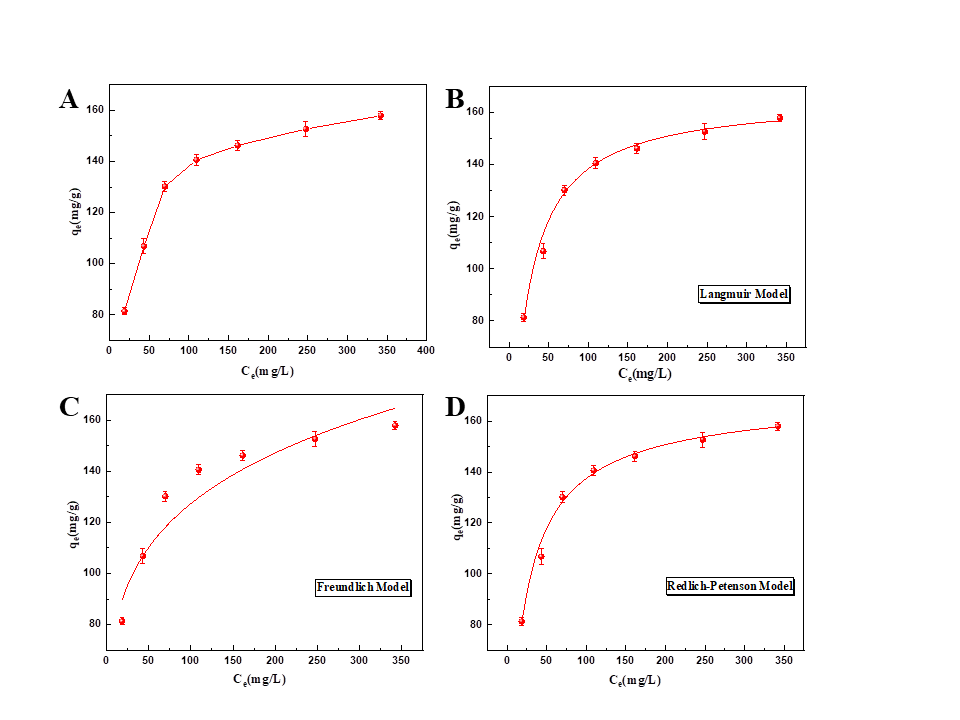
**

**Figure S3. Effect of the initial BB3 concentration on the adsorption capacity of UiO-66-NH_2_ at 298 K (A); Langmuir (B), Freundlich (C) and Redlich Peterson models (D).**

**Table S1. Kinetic parameters for the adsorption of BB3 on UiO-66-TLA.**

| **Adsorbent** | ***q*_e,exp_** | **Pseudo-first-order** | | | |  | **Pseudo-second-order** | | | | |  | **Intra-particle diffusion** | | | |  |
| --- | --- | --- | --- | --- | --- | --- | --- | --- | --- | --- | --- | --- | --- | --- | --- | --- | --- |
|  |  | *q*_e,cal_ | *k*_1_ (10^-2^) | *R*^2^ | *SSE* |  | *q*_e,cal_ | *k*_2_ (10^-3^) | *k*_2_*q*_e_ | *R*^2^ | *SSE* (10^-4^) |  | *k*_id_ | *C* (10^2^) | *R*^2^ | *SSE* |  |
| **UiO-66-TLA** | 225.7614 | 101.4281 | 3.016 | 0.89058 | 3.4202 |  | 7.1778 | 4.35 | 0.0312 | 0.99916 | 4.9553 |  | 4.9856 | 1.6938 | 0.9327 | 6.9942 |  |
| *q*_e, exp_ (mg g^-1^), *q*_e, cal_ (mg g^-1^), *k*_1_ (min^-1^), *k*_2_ (g mg^-1^ min^-1^), *k*_id_ (mg g^-1^ min^1/2^), *C* (mg g^-1^) | | | | | | | | | | | | | | |  |  |  |

**Table S2.** **Adsorption isotherm constants for adsorption of BB3 on UiO-66-TLA.**

| **Adsorbent** | **Temperature** | **Langmuir** | | |  | **Freundlich** | | |  | **Redlich Peterson** | | | |
| --- | --- | --- | --- | --- | --- | --- | --- | --- | --- | --- | --- | --- | --- |
|  |  | *q*_max_ | *K*_L_ | *R*^2^ |  | *K*_f_ | *n* | *R*^2^ |  | *a* | *b* | *g* | *R*^2^ |
| **UiO-66-TLA** | **298 K** | 283.8230 | 0.0433 | 0.8590 |  | 61.0314 | 3.6234 | 0.8364 |  | 0.9013 | 2.21*10^-7^ | 2.4172 | 0.9938 |
|  | **313 K** | 258.2467 | 0.06594 | 0.7052 |  | 69.4548 | 4.0668 | 0.9573 |  | 0.9905 | 3.46*10^-5^ | 1.63 | 0.9907 |
|  | **328 K** | 285.7342 | 0.0446 | 0.8623 |  | 64.8355 | 3.7270 | 0.9813 |  | 1.6536 | 1.12*10^-2^ | 1.10 | 0.9953 |
| *q*_max_ (mg g^-1^), *K_L_* (L mg^-1^), *K*_f_ (mg g^-1^), *a* (L g^-1^), *b* (J mol^-1^) | | | | | | | | | | | | | |

**Table S3.** **The elemental analysis results (wt. %) of UiO-66-NH_2_, UiO-66-TLA and UiO-66-TLA-BB3.**

| **Sample** | **C N** | **O** | **Zr Cl** |
| --- | --- | --- | --- |
| UiO-66-NH_2_ | 42.77 4.80 | 27.42 | 25.01 - |
| UiO-66-TLA | 41.16 4.92 | 21.23 | 32.69 - |
| UiO-66-TLA-BB3 | 45.07 5.78 | 23.45 | 25.51 0.19 |

**Table S4. Adsorption isotherm constants for adsorption of BB3 on UiO-66-NH_2._**

| **Adsorbent** | **Temperature** | **Langmuir** | | |  | **Freundlich** | | |  | **Redlich Peterson** | | | |
| --- | --- | --- | --- | --- | --- | --- | --- | --- | --- | --- | --- | --- | --- |
|  |  | *q*_max_ | *K*_L_ | *R*^2^ |  | *K*_f_ | *n* | *R*^2^ |  | *a* | *b* | *g* | *R*^2^ |
| **UiO-66-NH_2_** | **298 K** | 165.9745 | 0.05018 | 0.9951 |  | 48.5567 | 4.777 | 0.9112 |  | 8.8945 | 0.05956^-7^ | 0.9815 | 0.9945 |
| *q*_max_ (mg g^-1^), *K_L_* (L mg^-1^), *K*_f_ (mg g^-1^), *a* (L g^-1^), *b* (J mol^-1^) | | | | | | | | | | | | | |
